# 

**Published:** 1868-07

**Authors:** 


### Books and Pamphlets Received.

Lessons in Physical Diagnosis. By Alfred L. Loomis, M. D. New York: Robert M. DeWitt, publisher, No. 13 Frankfort street.

Catalogue and Announcement of the Medical Department of the University of Pennsylvania. Of the Missouri Medical College. Of the Bellevue Hospital Medical College. Of the Medical Department of the Washington University. Of the Medical Department of the Williamsville University. Of the Albany Medical College. Of the Annual Announcement of the University of New York.

A Medical Report upon the Uniform and Clothing of the Soldiers of the United States Army, Surgeon-General's Office 15th April, 1868.

### INDEX.

### A.

Abbott, Frank W., M. D. Review of Dr. C. C. F. Gay's article upon Uterine Surgery ... 347
Abbott, Frank W., M. D. Sources of Muscular Power, ... 293
Abstract of the Proceedings of the Buffalo Medical Association, 9, 41, 81, 137, 153, 174, 211, 256, 329, 369, 409, 453.
Academy of Medicine and Homœopathists, 181
Activity of the Skin in the Absorption of Medicine, ... 138
Acute Inflammation of the Psoas Magnus Muscle. By J. W. Grosvenor, M. D., ... 161
Address delivered before the Erie County Medical Society. By J. R. Lothrop, M. D., ... 241
Address delivered before the Graduating Class of the Buffalo Medical College. By J. F. Miner, M. D., ... 281
Alcohol as a Medicine. By Wm. M. Cornell, M. D., L. L. D., ... 106
Alcoholic Stimulants, Indiscriminate use of, in Disease, ... 64
Alimentation in Disease ... 421
Alleged Poisoning by Morphine, ... 121
American Medical Association, annual meeting of ... 362, 388
American Medical Association on Medical Literature, ... 39
American Medical Association, transactions of, ... 120
American Medical Association, Prize Essay for 1868, ... 200
American Journal of Obstetrics ... 408
Amputations at the knee-joint, ... 436
Anti-periodic, Liquor Ferri Persulphates as an, ... 219
Anuerism of the Right Subsclavian Artery, treated by direct pressure upon the Ateria Innomenata, ... 191
Appointment in the U. S. Navy, ... 368
Arsenic in the treatment of Pulmonary Phthisis, ... 430
Art of prescribing, ... 20
A Section on the Etiology of Uterine Diseases. By H. M. Congar, M. D., ... 81
Asiatic Cholera, treatment of. By Henry Nichell, M. D., ... 329
Association of Medical Superintendents of American Institutions for the Insane, annual meeting of ... 444
Association, New York State Medical, sixty-first anniversary, ... 266, 309
Atlantic Monthly, ... 237
Atomized Inhalations in Diseases of the Air Passages and Lungs. By Wm. M. Cornell, M. D., L. L. D., ... 61

### B.

Baxter, Surgeon J. H., promotion of, ... 38
Bichloride of Methylene as an anaesthetic, 359
Blisters in the treatment of Typhoid Fever, 71
Blodgett, A. C., M. D. Case of poisoning by Oil of Tansy, ... 136
Books and Pamphlets received, 40, 78, 119, 160, 238, 280, 327, 367, 406, 448, 483.
Boston School of Medical Specialties, ... 158
Bromide of Potassium in Epilepsy, ... 28
Bromide of Potassium, new property of, ... 328

### C.

Cæsarian Section. Mother and Child both saved, ... 353
Causes of the Formation of Cataract. By Alfred Mooren, M. D. Translated from the German, by C. F. A. Nichell, M.D., 214
Clinical Remarks upon Surgical Cases, at the Buffalo General Hospital. By J. F. Miner, M. D., ... 129, 172, 208, 341, 384
Clitoris, excision of, ... 16
Close of Volume VII, ... 476
Commencement Exercises of the Buffalo Medical College, ... 277
Commencement of new volume, ... 30
Congar, H. M., M. D. A Section on the Etiology of Uterine Diseases, ... 81
Consanguinous Marriages, ... 39
Contents of present number, ... 120
Cornell, Wm. M., M. D., L. L. D. Alcohol as a Medicine, ... 106
Cornell, Wm. M., M. D., L. L. D. Atomized Inhalations in Diseases of the Air Passages and Lungs, ... 61
Correspondence—Dental Surgery, ... 261
Correspondence—Letter from John G. Meacham, M. D., ... 259
Correspondence—Removal of a Neuromatous Tumor. By George D. Slocum, M. D., ... 308
Correspondence—Reply to a Review of Dr. Paul F. Eve's contributions to the History of Hip-joint Amputations, ... 257
Counter irritants, use of ... 392
Course and effects of Syphilis when untreated by any remedy, ... 224
Crew's Mustard Plaster, ... 408
Crystaline Lens, reproduction of, ... 112

### D.

Death from swallowing two ounces of Chloroform, ... 113
Death reported from inhalation of Ether, ... 140

Diarrhœa of Enteric or Typhoid Fever, ... 68
Diarrhœa, treatment of Infantile, ... 114
Dilator, Gentian Root as a, ... 229
Disease, alimentation in, ... 431
Doctors, puffing of in newspapers, ... 232
Doses and Actions of Medicines, ... 475
Druggists Renewing Prescriptions, ... 478

### E.

Empyema, treatment of in children ... 402
Entropium. By J. F. Miner, M. D., ... 384
Epileptics, legal responsibility of, ... 182
Epilepsy, Bromide of Potassium in, ... 28
Erie County Medical Association. Address to the—by J. R. Lothrop, M. D., ... 241
Erie County Medical Association, annual meeting of, ... 240
Erie County Medical Association, semi-annual meeting of, ... 439
Excision of the Clitoris, ... 16
Excision of the Median, Musculo-Spiral and Ulner Nerves. By J. F. Miner, M. D., ... 427
Experiments to prove that the Capillaries of the Lungs do not anastomoze ... 317
Extirpation of the Uterus, by a mistake for an Ovarian Tumor, ... 110
Extracts from a European Letter ... 71
Eve, Prof Paul F. Reply to a Review of Contributions to the History of Hip-joint Amputations, ... 257
Every Saturday ... 237
Experiments Showing Vegetable organisms in Human Blood, ... 467

### F.

Fibris Nigra, recent outbreak of in Dublin, 397
Fistula in Ano. Clinical Remarks upon. By J. F. Miner, M. D., ... 208
Florence Nightingale. Ward in King's College Hospital, ... 473
Forms of continued Fever and their definition, ... 400
Fractures, Simple and Compound, of the Leg, treated with Suspension Splints, ... 262

### G.

Gay, C. C. F., M. D. Placenta Previa, ... 369
Gay, C. C. F., M. D. Uterine Displacements and their Surgical Treatment, ... 418
Gay, C. C. F., M. D. Uterine Surgery, ... 299
Gay, C. C. F., M. D. Vesico Vaginal Fistula ... 103, 131, 166
Genesee County Medical Society, ... 440
Gentian Root as a Dilator, ... 229
Grosvenor J. W., M. D. Acute Inflammation of the Psoas Magnus Muscle, ... 161

### H.

Hahnemann Medical College, ... 408
Half-Yearly Compendium Medical Sciences, ... 159
Hernia, Operation for Strangulated. By J. R. Lothrop, M. D., ... 1
Hernia, Operation for Strangulated Femoral. By J. F. Miner, M. D ... 208
Homœopathists and the Academy of Medicine, ... 159
Homœopathy and the University of Michigan, ... 356
Homœopathy Putting on Airs ... 408
Hospitals and Dispensaries, ... 484

### I.

Indiscriminate use of Alcoholic Stimulants in Disease ... 64
Induction of Premature Labor, ... 478
Infantile Diarrhœa, treatment of, ... 114
Inflammation, general and local treatment of. By J. F. Miner, M. D., ... 201
Immature Observations, ... 433
Inoculation of Tubercle, ... 142
Instrumental Diagnosis. Sphymography, ... 462
International Medical Congress, ... 80, 146

### J.

Jones, W. W., M. D. Life Insurance, ... 14

### K.

Knee-joint Amputations, ... 436

### L.

Law-suits upon shares, ... 157
Lee, Prof. Charles A., Resignation of, ... 199
Legal Responsibility of Epileptics, ... 182
Life Insurance. By W. W. Jones, M. D., ... 14
Liquor Ferri Persulphates, as an antiperiodic, ... 219
London Hospitals, Resections in, ... 226
London Lancet, ... 483
Long Island Medical College ... 200
Locomotor Ataxy, treated with Nitrate of Silver, ... 230
Lothrop, J. R., M. D, Annual Address before the Erie County Medical Society, ... 241
Lothrop, J. R., M. D. Strangulated Hernia, ... 1

### M.

Magnetic Somnambulism, ... 24
Materia Medica, General Therapeutics and Pharmacy, ... 220
Meaham, John G., M. D., ... 259
Medical Colleges, ... 115
Medical College in Detroit, Mich., ... 408
Medicine as a Business, and Business in Medicine, ... 442
Memorandum on the nature and mode of Propagation of Phthisis, ... 187
Milk of Syphlatic Nurses, ... 474
Miner, J. F., M. D. Annual Address to the Graduating Class of the Buffalo Medical College, ... 281
Miner, J. F., M. D. Clinical Remarks upon Surgical Cases at the Buffalo General Hospital, ... 129, 172, 208, 341, 384
Miner, J. F., M. D. General and Local Treatment of Inflammation, ... 201
Miner, J. F., M. D. Excision of the Median, Musculo-Spiral and Ulnar Nerves, ... 427
Mooren, Alfred, M. D. Causes of the Formation of Cataract. Translated from the German, by C. F. A. Nichell, M. D. ... 214
Monthly Medical Reprint, ... 484

### N.

Nerves, Excision of the Median Musculo-Spiral and Ulnar. By J. F. Miner, M.D., 427
New Anæsthetic. Bichloride of Methylene, ... 359
New Medical Journal, ... 328

New Medical Works, 200
New mode of Vaccination, 234
New Method of Preserving the Dead, 80
New Property of Bromide of Potassium, 328
New Sydenham Society, 38
New Sydenham Society, Publications of, 448
New York Academy of Medicine and Mr. Hoff, 23
New York Academy of Medicine. Consultation with Homœopathist, 193
Nichell, Henry, M. D. Treatment of Asiatic Cholera, 329
Nitrate of Silver in the Treatment of Locomoter Ataxy, 230

### O.

Obituary. John Mason Warren, M. D., 31
Obituary. John C. Mattison, M. D., 408
Obituary. M. Eugene Shaw, M. D., 158
Obituary Notices, 79
Obituary. Thomas C. Brinsmade, M. D., 447

### P.

Personal Items, 482
Phthisis. Memorandum on the Nature and Mode of Propagation, 187
Phthisis, Value of Arsenic in the Treatment of, 430
Placenta Previa, 369
Poisoning by Morphine, 121
Poisoning by Oil of Tansy. By A. C. Blodgett, M. D., 136
Preparation and Publication of the Medical and Surgical History of the War, 443
Prescribing, art of, 20
Prescriptions, renewal of, by Druggists, 437
Prescriptions, written, 73
Psoas Magnus Muscle, Acute Inflammation of. By J. W. Grosvenor, M. D., 161
Puffing of Doctors by the Newspapers, 232
Pure Bourbon Whisky, 482

### R.

Raid on the Uterus, 22
Remarkable Infant. J. Myrtle Corban, 459
Recent outbreak of Fibris Nigra in Dublin, 397
Renewal of Prescriptions by Druggists, 437
Report of the Scientific Committee appointed to investigate the Physiological and Therapeutical effects of the Hypodermic Method of Injection, 78
Reproduction of the Crystaline Lens, 112
Resections in the London Hospitals, 226
Review—Aikins' Science and Practice of Medicine, 32
" Annual Report of the Surgeon General U. S. A., 1867, 279
" Bartholow on the Principles and Practice of Disinfection, 76, 364
" Beard & Rockwell. Medical Use of Electricity, 118
" Bedford. Principles and Practice of Obstetrics, 367
" Bedele's Materia Medica, 480
" Bennett's Principles and Practice of Medicine, 116
" Biennial Retrospect of Medicine and Surgery and the Allied Sciences, 198
" Biglow on Ununited Fractures, 77
" Bouchardett's Annual Abstract of Therapeutics, Materia Medica, Pharmacy and Toxicology, for 1867, 364
Review—Brand & Taylor's Chemistry, 119
" Carson's Synopsis of Materia Medica, 238
" Catalogue of the Army Medical Museum, 194
" Chambers on Indigestion, 479
" Circular No. 7. Amputations at the Hip-joint, 154
" Circular No. 5. Report on Epidemic Cholera, 36
" Code of Medical Ethics of the American Medical Association, 78
" Condie on Diseases of Children, 325
" Coventry on Spotted or Congestive Fever, 198
" Cullerier's Atlas of Venereal Diseases, 405
" Dalton's Human Physiology, 156
" Dickson. Studies in Pathology and Therapeutics, 
" Elliot's Obstetrical Clinic, 366
" Elmer's Physicians' Hand-Book, 1868, 196
" Erichson on Railway and other Injuries to the Nervous System, 78
" Eve. Contributions to the History of Hip-joint Amputations, 235
" Flint's Physiology of Man, 197
" Fuller on Diseases of the Lungs and Air Passages, 324
" Half-Yearly Compendium of Medical Science, 482
" Hartshorne's Principles and Practice of Medicine, 119
" Headland on the Action of Medicines in the System, 34
" Hewett on the Diseases of Women, 480
" Holden's Manual of Disections of the Human Body, 406
" Howard Damon on Neurosus of the Skin, 481
" Hufland's Art of Prolonging Life, 156
" Institutes of Medicine. By Martyn Paine, A.M., M.D., L.L.D., 481
" Jennings' Tree of Life, 153
" Lawson on Injuries of the Eye, 199
" Lindsay & Blackiston's Physicians' Visiting List, for 1868, 199
" Maudsly on the Physiology and Pathology of the Mind, 117
" Miller on the Causes of Exhausted Vitality, 238
" Morris on Shock after Surgical Operations and Injuries, 236
" Pennsylvania Hospital Report. Vol. 1, 326
" Peters on Asiatic Cholera, 77
" Prince. Plastic Surgery, 365
" Siegle on the Treatment of Diseases of the Throat and Air Passages, 279
" Stillé on Epidemic Meningitis, 238
" Stillé. Therapeutics and Materia Medica, 445
" Storer. "Is it I," 157
" Tanner. Signs and Diseases of Pregnancy, 405
" Thomas on Diseases of Women, 401
" Tobald. Chronic Diseases of the Larynx, 446
" Transactions of the American Medical Association. Vol. 18. 1867, 195
" Transactions of the Medical Society of the State of Kansas 
" United States Sanitary Commission, Memoirs. Edited by Austin Flint, 481

Review—Wales on Mechanical Therapeutics, 280
" West. Lectures on Diseases of Women, 237
" Wetmore. The Base of the Brain with Nerves emerging, 198
" Williams on Diseases of the Eye, Eye. Their Medical and Surgical Treatment, 118
" Wilson on Diseases of the Skin, 479
" Wright on Headaches 237
Richmond and Louisville Medical Journal, 368

### S.

Slocum, George D. Removal of a Neuromatous Tumor, 308
Skin, Activity of, in the Absorption of Medicines, 138
Somnambulism, Magnetic, 24
Sources of Muscular Power. By Frank W. Abbott, M. D., 293
Subsclavian Artery, aneurism of, treated by direct pressure upon the Ateria Innomenata, 191
Subcutaneous Injection, 484
Surgical Pathology and Therapeutics and Operative Surgery, 141
Sustaining the Board of Regents, 478
Suspension Splint for treating simple and compound fractures of the leg, 262
Suture of Nerves, 466
Syphilis, Course and effects of, when untreated by any remedy, 224
Storer, Prof. Horatio R. Lectures to Physicians, 37
Strong, P. H., M. D. Typhoid Fever. Is it arrestable? 41

### T.

Transactions of the American Medical Association, 120, 388
Transactions of the Buffalo Medical Association, 9, 41, 81, 137, 174, 211, 256, 329, 369, 409.
Transactions of the Erie County Medical Society, 240, 439
Transactions of the New York State Medical Society 266, 309
Treatment of Disease by Faith and Prayer instead of Medicine, 438
Tubercle, Inoculation of, 142
Typhoid Fever, Blisters in the treatment of, 114
Typhoid Fever, Diarrhœa in, 68
Typhoid Fever. Is it arrestable? By P. H. Strong, M. D., 41

### U.

University of Michigan vs. Homœopathy, 356
Use of Counter Irritants, 392
Use of Pepsine in Alimentation in Disease. By James S. Hawley, M. D., 449
Uterine Displacements and their Surgical Treatment. By C. C. F. Gay, M. D., 418
Uterine Surgery. By C. C. F. Gay, M. D., 299
Uterus. Extirpation of, by mistake for an Ovarian Tumor 110
Uterus, Raid on the, 22

### V.

Vaccination, New method of, 234
Value of Arsenic in the Treatment of Pulmonary Phthisis, 430
Vesico Vaginal Fistula. By C. C. F. Gay, M. D., 103, 131, 166
Vesico Vaginal Fistula, Operation for. By J. F. Miner, M. D., 384

### W.

Written Prescriptions, 73

